# Influence of emulsifiers on the characteristics of polyurethane structures used as drug carrier

**DOI:** 10.1186/1752-153X-7-66

**Published:** 2013-04-10

**Authors:** Alina Heghes, Codruta M Soica, Simona Ardelean, Rita Ambrus, Danina Muntean, Atena Galuscan, Dan Dragos, Daniela Ionescu, Florin Borcan

**Affiliations:** 1 Faculty of Pharmacy, “Victor Babes”, University of Medicine and Pharmacy Timisoara, 2nd E. Murgu Sq, Timisoara 300041, Romania; 2 Faculty of General Medicine, Pharmacy and Dental Medicine, “Vasile Goldis” University, 1st Feleacului Str, Arad 310396, Romania; 3 Department of Pharmaceutical Technology, University of Szeged, 6th Eotvos Str, Szeged H-6720, Hungary; 4 Faculty of Medicine, “Victor Babes” University of Medicine and Pharmacy Timisoara, 2nd E. Murgu Sq, Timisoara 300041, Romania; 5 Faculty of Dentistry, “Victor Babes” University of Medicine and Pharmacy Timisoara, 14th T. Vladimirescu Str, Timisoara 300173, Romania

**Keywords:** Hollow structures, Polyurethane, Hexamethylene diisocyanate, Zeta potential, TEWL, Mexameter

## Abstract

**Background:**

Emulsifiers have a significant role in the emulsion polymerization by reducing the interfacial tension thus increasing the stability of colloidal dispersions of polymer nanostructures. This study evaluates the impact of four emulsifiers on the characteristics of polyurethane hollow structures used as drug delivery system.

**Results:**

Polyurethane (PU) structures with high stability and sizes ranging from nano- to micro-scale were obtained by interfacial polyaddition combined with spontaneous emulsification. The pH of PU aqueous solutions (0.1% w/w) was slightly acidic, which is acceptable for products intended to be used on human skin. Agglomerated structures with irregular shapes were observed by scanning electron microscopy. The synthesized structures have melting points between 245-265°C and reveal promising results in different evaluations (TEWL, mexametry) on murine skin.

**Conclusions:**

In this study hollow PU structures of reduced noxiousness were synthesized, their size and stability being influenced by emulsifiers. Such structures could be used in the pharmaceutical field as future drug delivery systems.

## Background

Several varieties of structures (polymeric and metal nano- and micro-particles, liposomes, micelles, quantum dots, dendrimers, lipoproteins, nanotubs) were used as drug carriers in order to reduce drug metabolism, drug toxicity and to prolong *in vivo* drug activity [1]. The liver isoenzymes known as cytochrome P450 are involved in phase I of drug metabolism [2]. During a study of drug elimination pathways it was established that the cytochrome P450 is responsible for complete removal from the human body of more than a half of 400 drugs marketed in Europe and US [3]. Toxicity studies for polymer-methotrexate conjugate vs. free methotrexate on murine rheumatoid arthritis model indicate that the free compound starts to cause animals’death at the maximum tolerated dose and is highly toxic at LD50 level, while the polymer-methotrexate conjugate shows no toxicity at the same equivalent concentrations [4]. The drug carriers can prolong the *in vivo* drug activity, both by their controlled drug-release technology and a long-lasting target binding and rebinding mechanism [5].

Polyurethanes are commonly used in medical applications and their use continues to expand due to their versatility, biocompatibility and hemocompatibility. There are several types of PU, including the following: liquid PU for hollow-fiber devices, PU for dip-molding, PU coatings, biostable PU and thermoplastic PU [6]. Sivak WN [7] synthesized a novel PU drug delivery system based on lysine diisocyanate and glycerol using various tertiary amines and organometallic urethane as catalysts. The use of LDI-glycerol PU foams as drug carriers for the controlled release of 7-tert-butyldimethylsilyl-10-hydroxy-camptothecin (DB-67) revealed that such foams were capable of delivering therapeutic concentrations of DB-67 *in vitro* over an 11 weeks trial period [8].

The purpose of the present investigation was to develop aliphatic PU structures with a diameter in the range of 100-300 nm in the absence of a catalyst. We also studied the effect of four emulsifiers on the structures’ size and stability and also their noxiousness on murine skin model.

## Results and discussion

The development of novel targeted nano-polymers in the drug delivery field is currently a research topic of high interest [9,10]. In our research the main aim was to avoid the potentially toxic additional raw materials. Only aliphatic compounds were used even if it is well-known that these compounds lead to final products of poor physical and mechanical properties [11]. In this synthesis the presence of a chains’ initiator was not necessary and the reaction went without catalyst. Unlike previous studies [12] a single surfactant has been used instead of a mixture (lipophilic or hydrophilic) and only in a small amount.

The pH values of PU solutions were determined using a Schott TitroLine at 25°C as described in the literature [13]. The measurements were made in triplicate and data were expressed as mean and standard deviation. The obtained samples show slightly acidic pH values (5.88±0.11 for sample PuS-1, 6.07±0.09 for sample PuS-2, 5.98±0.16 for PuS-3 and 6.24±0.09 for sample PuS-4). The above pH values are appropriate for products intended for skin application considering that skin pH is around 5.80 for men and around 5.54 for women [14]. Variable skin pH values were reported in the literature, all in the acidic domain, but varying from 4.0 to 7.0 depending on the purpose of using [15]. These slightly acidic solutions are acceptable for dermal applications because they do not cause dry skin as a regular soap does and also maintain the normal skin microflora.

The results obtained by using Zetasizer Nano ZS indicate that PU structures are relatively homogeneous and their size ranges from nano- to micro-scale (Table 1). The increase of the emulsifier amount would most probably reduce the structures’ size below 100 nm (the limit between the two scales) because emulsifiers produce lower interfacial tension and increase the stability of colloidal dispersions, therefore avoiding aggregates’ formation.

**Table 1:** The PU structures characteristics obtained by Zetasizer Nano ZS

**Sample code**	**Emulsifier**	**Particle size (nm)**		**Zeta potential (mV) mean ± SD**
		**Mean ± SD**	**Polydispersity index**	
PuS-1	Cremophor EL	164 ± 12	0.2	33.9 ± 5.0
PuS-2	Cremophor A6	201 ± 19	0.5	37.5 ± 4.4
PuS-3	Cremophor A25	298 ± 11	0.4	23.0 ± 4.8
PuS-4	Cremophor RH40	379 ± 23	0.3	29.7 ± 3.1

Zeta potential values are used to predict particles’ stability, stable particles presenting a zeta potential more negative than -30 mV or more positive than +30 mV [16]. All this considered, the PU structures obtained in the first two experiments (using Cremophor EL and Cremophor A6 as emulsifiers) are considered the most stable products.

**Figure 1:**
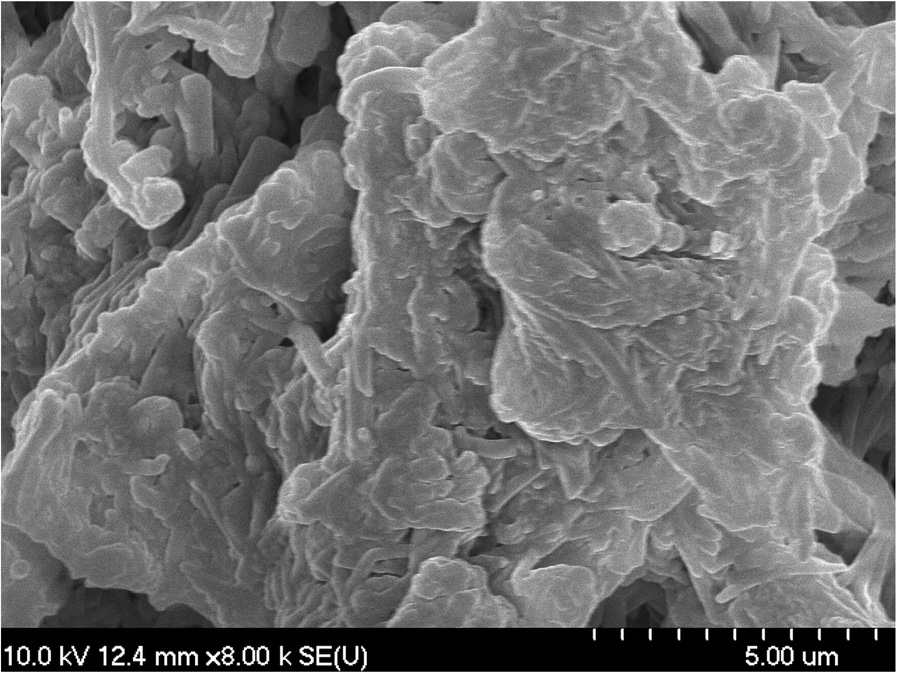
SEM image of PuS-1 sample.

**Figure 2:**
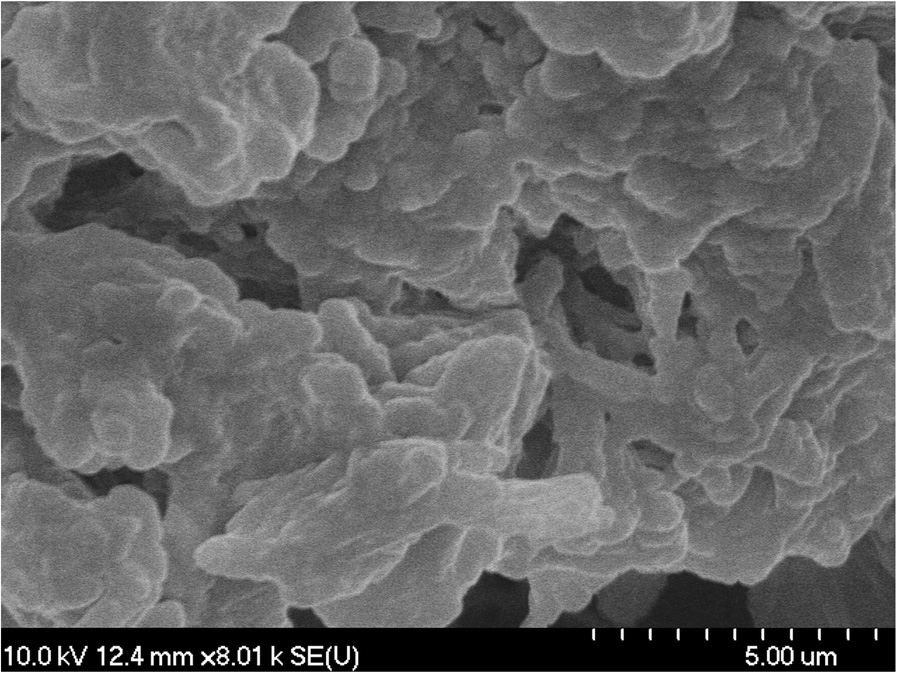
SEM image of PuS-2 sample.

**Figure 3:**
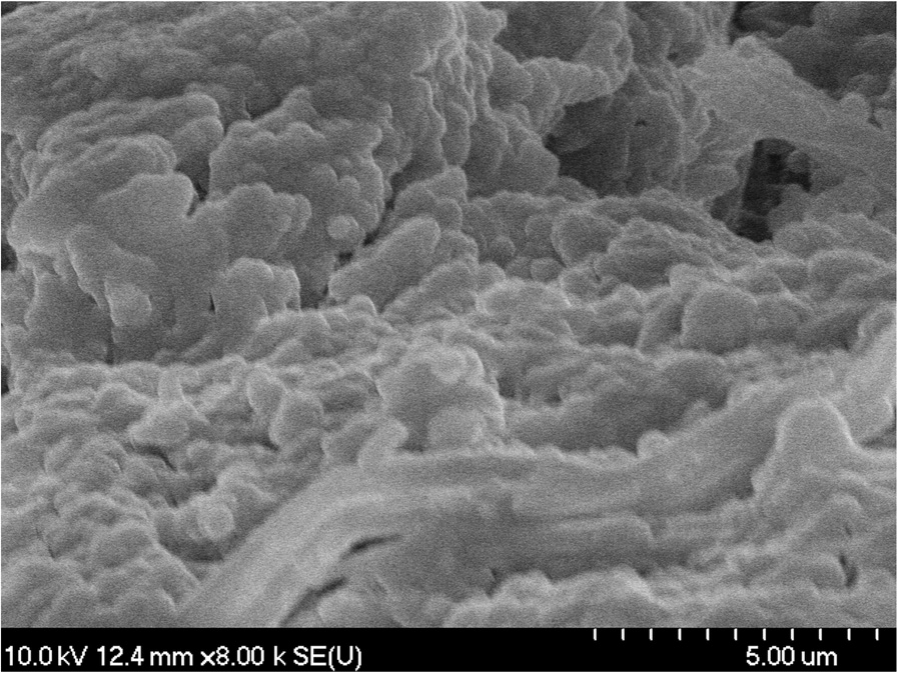
SEM image of PuS-3 sample.

**Figure 4:**
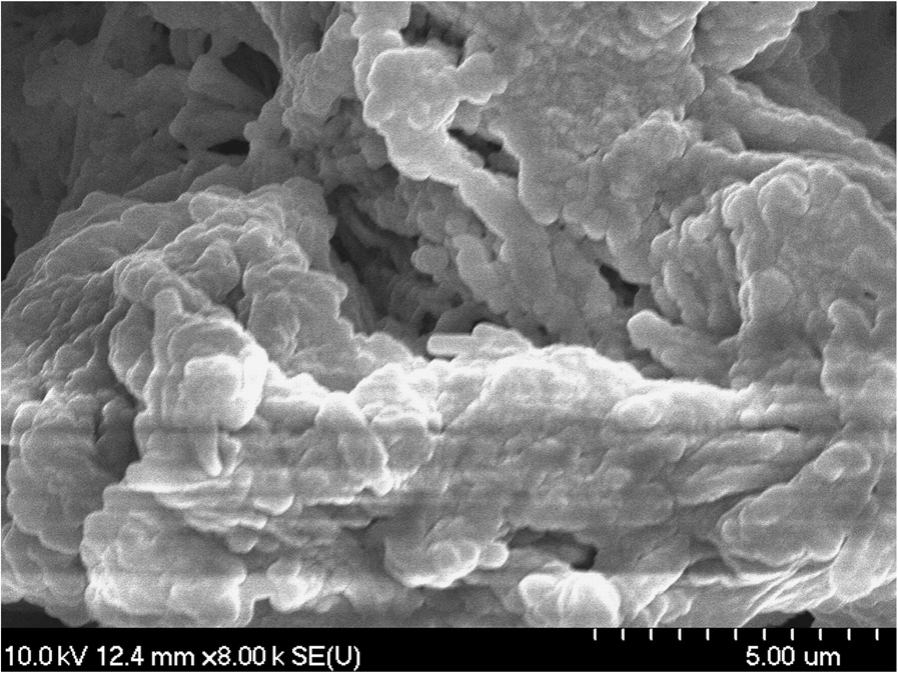
SEM image of PuS-4 sample.

The presence of macromolecular aggregates with irregular shapes was noticed using scanning electron microscopy (SEM). Irregular amorphous-crystalline nature of the samples can also be seen in SEM images (Figures 1, 2, 3, 4). The non-spherical shape of the PU structures affect their flowing properties and might also influence their targeting ability [17]. SEM pictures revealed a non-porous material which represents an advantage for a drug carrier in terms of protection of its load. Non-agglomerated structures cannot be successfully synthesized probably due to the small amount of emulsifier which affects the structures’ stability and tendency to pack [18].

**Figure 5:**
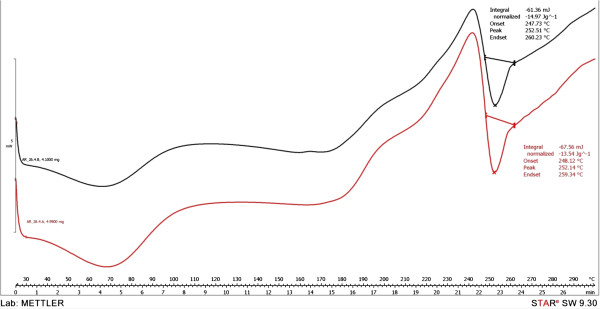
DSC of PuS-1 sample (black) and PuS-2 sample (red).

**Figure 6:**
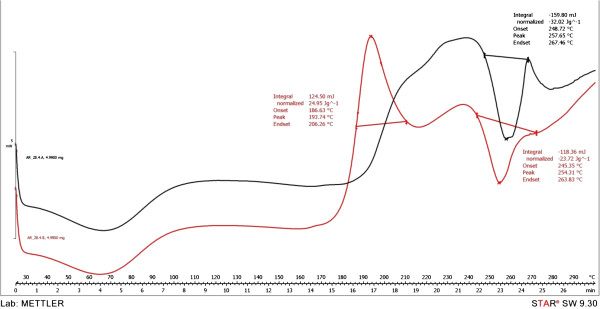
DSC of PuS-3 sample (black) and PuS-4 sample (red).

At the glass transition temperature, Tg, the molecules have enough energy to partially overcome the intermolecular forces, and have more freedom of movement [19]. The presence of the PU structures glass transition is noticed on the DSC curves around 65°C, which reveals the predominant crystalline nature of the polymer [20]. A crystallization exothermic peak (+124.5 mJ) was recorded at 193.74°C in the case of PuS-4 sample. Further heating above the crystallization temperature results in an endothermic peak which corresponds to the melting of the crystalline region [21]. The melting points of the synthesized PU structures were between 245-265°C (Figures 5, 6). The thermal behaviour survey of the obtained PU structures has revealed that Cremophor-type emulsifiers did not significantly influence the melting process.

**Figure 7:**
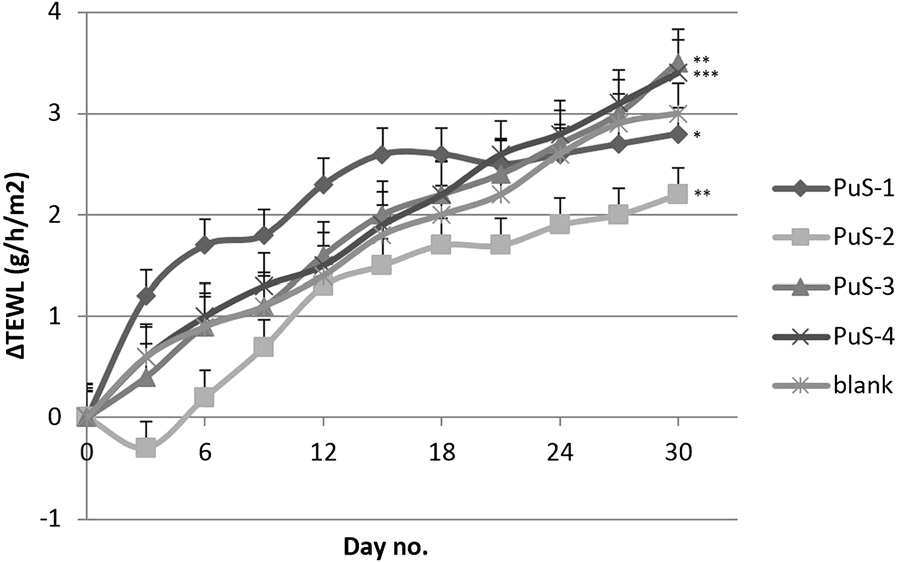
Evolutions of transepidermal water loss.

TEWL was measured using a non-invasive procedure in order to detect any skin barrier disorder. In the first day of the experiment, all mice exhibited TEWL values between 0-10 g/h/m^2^, which is the specific range for a healthy skin, values that were used as reference [22]. No significant differences were noticed between animal groups or between the first and last day of the experiment (Figure 7). The best results were obtained in case of the mice treated with PuS-1 and PuS-2 creams (TEWL differences below 3 g/h/m^2^ in 30 days, values which were also recorded in the case of blank cream application).

**Figure 8:**
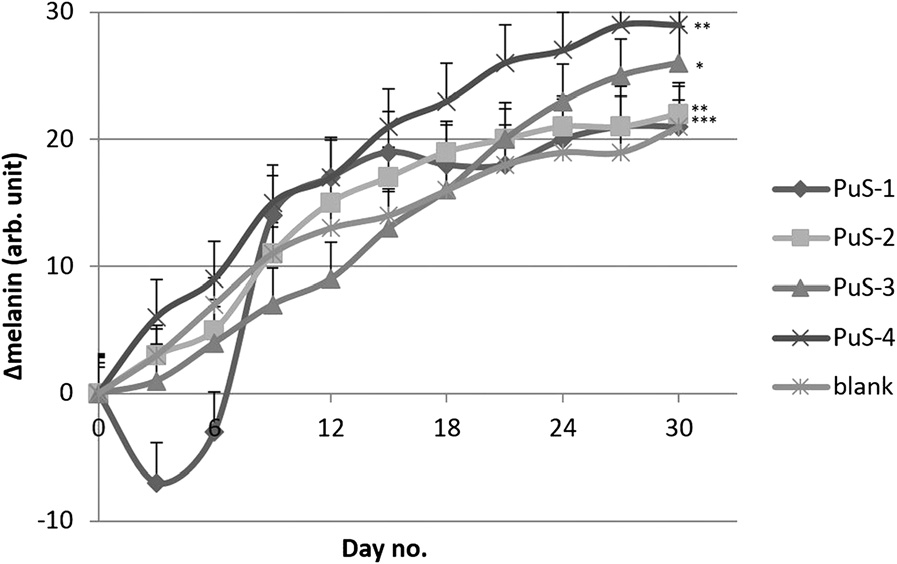
Evolutions of melanin values.

Found in the basal layer of the epidermis, melanocytes produce melanin which determines skin colour [23]. Studies concerning the melanin content of nude mice skin were developed in order to establish the human malignant melanomas’ progress [24], radioimmunotherapy efficacy against experimental human metastatic melanoma [25] or the effect of skin pigmentation in bioluminescent imaging [26]. It is well-known that skin often reacts to external agents by modifying the constitutive pigmentation pattern [27]. In this study, the melanin content was investigated using mice skin, which presents extremely close values (maximum range of variation was 10 melanin arb. units in the first day of the experiment). The melanin evolution (Figure 8) presents an increase for all mice (whether they were treated with blank cream or cream with PU structures); it is pertinent to mention that in the case of mice treated with PuS-1 and PuS-2 creams the increase of melanin content was not so pronounced.

**Figure 9:**
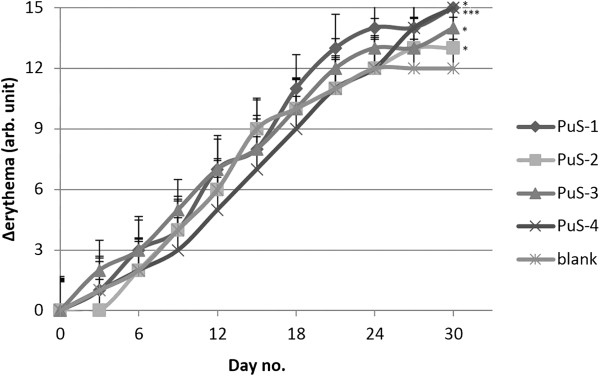
Evolutions of erythema values.

Erythema (redness of the skin) caused by expansion and congestion of the capillaries is usually a sign of inflammation or infection [28]. Irritant compounds such as surfactants have been extensively studied in the last decade and it has been noticed that erythema values can be correlated with their irritation potential [29]. In this experiment, the lowest change of the erythema (local haemoglobin) values was noticed in case of the mice treated with blank cream and PuS-2 cream respectively, which recommends the latter as the transdermal carrier with the lowest toxicity (Figure 9).

## Conclusions

In this research PU structures were synthesized using a well-known procedure, the interfacial polyaddition combined with spontaneous emulsification. HMDI in acetone was used as isocyanate component and a mixture of MEG, 1,4-BD and PEG 200 in water was used as hydroxylic component. Four different emulsifiers were used and the synthesis was done without any chain initiator or catalyst. The PU structures showed a good stability, melting points between 245-265°C, and sizes between 150-400 nm. The PU structures were embedded in a cream for topical applications on murine skin. Evolution of TEWL, melanin content and skin erythema were recorded for a period of 30 days. The comparative analysis revealed that PU structures based on Cremophor A6 present the best toxicological profile and can be used as drug carriers for therapeutic purposes.

## Methods

### Raw materials

Hexamethylene diisocyanate (HMDI), polyethylene glycol (PEG 200) and acetone were obtained from Merck (Germany). Emulsifiers (Cremophor EL, Cremophor A6, Cremophor A25, and Cremophor RH40) were kindly donated by our colleagues from University of Szeged (Hungary). Mono-ethylene glycol (MEG) was purchased from Lach-Ner s.r.o. (Czech Rep.) while 1,4-butanediol (1,4-BD) was purchased from Carl Roth GmbH (Germany). All substances were used as received.

### Synthesis protocol

The influence of the aqueous components ratio [30], isocyanate component [31], and influence of stirring speed [32] were already studied by our team in order to optimize the structures sizes and stability.

**Figure 10:**
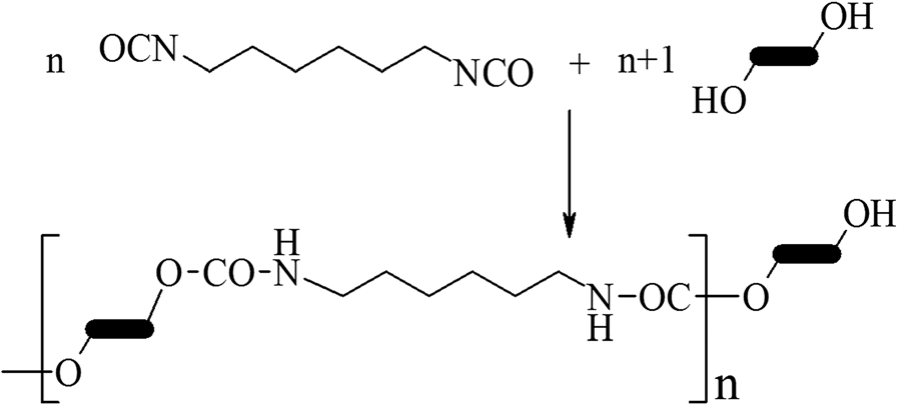
Reaction of PU structures synthesis.

The polyaddition reaction between HMDI and diol-polyether mixture for the PU structures synthesis follows the reaction from Figure 10.

Isocyanate and hydroxyl components were used in a 1:1.1 molar ratio in order to reduce the amount of secondary products (amines) and to ensure an easier washing of products by using distilled water.

PU structures synthesis procedure was already described in the literature [12,31] and involves the following steps:

1. Phases preparation – HMDI in acetone 0.5 mM solution (organic phase) was heated at 40°C; MEG, 1,4-BD and PEG 200 (1:1:2, molar ratio) in distilled water 0.5 mM solution (aqueous phase) was mixed with different emulsifiers and heated at 40°C.

2. The organic phase was injected into the aqueous phase at 40°C under magnetic stirring (500 rpm). The stirring was maintained for 4 hours at 40°C in order to ensure the maturation of the PU structures walls, even if the structures were formed in the first three minutes.

3. Acetone and a part of water were removed by slow evaporation in the oven, keeping the suspensions as thin layers (approx. 3 mm) in Petri dishes at 60°C for 12 hours. Products were purified by three cycles of centrifugation and dispersion in a mixture of water-acetone (1:1, v/v) in order to eliminate possible secondary products or unreacted raw materials.

The previously described procedure was repeated four times with different emulsifiers, which were chosen in order to study their effects on the PU characteristics (samples: PuS-1 with Cremophor EL, 1.5 ml; PuS-2 with Cremophor A6, 0.2 g; PuS-3 with Cremophor A25, 0.2 g; PuS-4 with Cremophor RH40, 0.2 g). The samples were carefully dried and the pH of PU aqueous solutions was measured at the same concentration.

### Physical and chemical characterization

The shape and morphology of the final PU structures were examined with a Hitachi 2400S SEM as already described in the literature [33].

Thermal analysis was carried out using a Mettler-Toledo 821e instrument between 30-300°C because most of the urethane groups (-NH-COO-) are stable in this temperature range [34].

Particles size and charge were measured using a Zetasizer Nano series equipment Nano-Zs, Malvern Instruments. Samples were diluted in distilled water at a ratio of 1:5000 v/v. The measurements were carried out in duplicate for each sample.

*In vitro* characterization of similar PU structures was already done by our team by testing of mesenchymal stem cells (MSCs) viability. There were obtained good results after 48 hours based on the Alamar Blue test [31].

### Animals

Ten CD1Nu/Nu eight weeks old female mice were purchased from Charles River (Sulzfeld, Germany). The protocol followed all National Institute of Animal Health (NIAH) rules: during the experiment animals were maintained in standard conditions of 12 hours light-dark cycle, food and water *ad libitum*, 24±1°C, humidity above 55%. Mice were divided into five equal groups (2 mice for blank cream and each PU structures type, respectively).

### Evaluation of skin parameters

PU structures were included in a cream prepared as previously described in the literature by Pavicic T et al. [35] in order to observe how they affect skin parameters. The blank cream used was an oil-in-water emulsion containing water, hydrogenated polydecene, steareth-2, cetearyl alcohol, phenoxyethanol, methylparaben, diazolidinyl urea and disodium EDTA; the other formulations contain the same ingredients and additionally 0.2% PU structures. The cream was stretched with a gentle massage till it was totally absorbed into the back skin every third day (1 ml / application).

After each application, determination of skin parameters was performed within 30 minutes. All the measurements were carried out according to the published guidelines [36] with a Multiprobe Adapter System (MPA5) from Courage&Khazaka Electronics, Germany, equipped with a Tewameter®TM300 probe and a Mexameter®MX18 probe. All measurements were done at the same moment of the day, by the same operator, in a narrow range of temperature (24±1°C) and air humidity (45±3%).

The nude mice have been used in many studies in immunology, pathology, genetics, virology, parasitology, endocrinology, dermatology, radiology and many other areas [37]. Nude mice are not bald but instead show an 'abortive' reduced hair growth on different sites of the integument [38]. In this research, CD1Nu/Nu female mice skin was chosen to study newly synthesized PU structures’ noxiousness; their skin is sensitive to experimentally induced infections or to other diseases, the lack of hair being the most significant predisposing factor [39].

Skin is the soft outer layer of humans and most animals. The skin plays a crucial role in protecting the body against pathogens [40]. Another main role is the function as a barrier to water permeation, a phenomenon of normal transfer of water through the stratum corneum into the atmosphere, known as transepidermal water loss (TEWL), which represents part of insensible water loss [41]. TEWL may increase due to disruption of the skin barrier (wounds, scratches, burns, exposure to solvents or surfactants, extreme dryness) and is affected by humidity, temperature, season and moisture content of the skin (hydration level) [42].

Transepidermal water loss (TEWL) was reported to predict the irritation potential of a given compound, e.g. a notable increase of TEWL, meaning a significant reduction of the skin barrier function, was recorded when sodium laurylsulfate (SLS) was applied on the skin [43]. In the last years, TEWL studies focused on the benefits of phytocompounds used in cosmetology [44] and of the anti-aging skin products [45]. In this research, the measurements with Tewameter®TM300 probe were accomplished following a procedure previously described in the literature [46]: the probe was recalibrated in the first day of the trial according to the manufacturer’s indications [47] while room humidity and temperature were continuously monitored and kept in a narrow range of values.

Mexametry is an easy, quick and inexpensive technique used to determine the two components mainly responsible for the colour of the skin: melanin and haemoglobin (erythema) [48]. In this study, a Mexameter®MX18 probe with three light-emitting diodes for green, red and infrared light was used. Instrumental evaluations of skin colour were performed after each cream application (every third day). The probe was maintained on the application spot until the device software displayed the measured values on the computer screen (1-3 seconds).

All measurements using the Courage&Khazaka MPA5 device were done in triplicate and data were expressed as mean and standard deviation of the differences between the current day and the first day of the trial for the same mouse.

Paired Student’s t tests or One-way Anova followed by Bonferroni’s post-tests were used to determine the statistical difference between different experimental and blank groups: *, ** and *** indicate p<0.05, p<0.01 and <0.001.

## Competing interests

This work was financial supported by The National Research Council from Romania (UEFISCDI): research project PNII-PD-586/2010 (contract no. 110 / 12.08.2010).

## Authors' contributions

CMS, DD and FB have synthesized and purified the PU structures. RA has carried out SEM and DSC analyses. AH, AS, DM, DI and AG have conceived the study, and participated in its design and coordination and helped to draft the manuscript. All authors have read and approved the final manuscript.
